# Risk of Skin Cancer in Patients with Psoriasis: Single-Center Retrospective Study Comparing Anti-TNFα and Phototherapy

**DOI:** 10.3390/jcm13092452

**Published:** 2024-04-23

**Authors:** Emanuele Trovato, Martina Dragotto, Eugenio Capalbo, Alessandra Cartocci, Pietro Rubegni, Laura Calabrese

**Affiliations:** 1 Dermatology Unit, Department of Medical, Surgical and Neurological Sciences, University of Siena, 53100 Siena, Italy; emanuele.trovato@unisi.it (E.T.); m.dragotto@student.unisi.it (M.D.); pietro.rubegni@unisi.it (P.R.); 2 Department of Medical Biotechnology, University of Siena, 53100 Siena, Italy; 3 Dermatologia, Dipartimento di Medicina e Chirurgia Traslazionale, Università Cattolica del Sacro Cuore, 00168 Rome, Italy

**Keywords:** psoriasis, non-melanoma skin cancers, basal cell carcinoma, squamous cell carcinoma, biologic agents, anti-TNFα, phototherapy, nb-UVB

## Abstract

**Background:** The risk of developing non-melanoma skin cancers (NMSCs) in patients with psoriasis is highly debated, and, to date, there is no unambiguous consensus opinion. Psoriasis is known to be related to an increased likelihood of other comorbidities such as psoriatic arthritis, obesity, metabolic syndrome, depression, and cardiovascular disease. Regarding cancer risk, previous studies have reported a greater tendency for the development of cutaneous T-lymphomas and colon, breast, kidney, and lung cancers. Furthermore, data from network meta-analyses have shown that patients with psoriasis have a higher risk of developing squamous cell carcinomas (SCCs) and/or basal cell carcinomas (BCCs). Multiple factors may contribute to the development of NMSCs in psoriatic patients, ranging from immunosuppression induced by biologic agents to previous phototherapy. However, the extent to which each factor may impact this risk has not been entirely assessed. The aim of this study was to evaluate the risk of developing NMSCs in patients with psoriasis observed for at least 5 years, by directly comparing patients only treated with phototherapy and patients treated with anti-tumor necrosis factor α (TNFα) agents, naive to other systemic treatments or phototherapy. **Methods:** We conducted a single-center retrospective study at Siena University Hospital, Italy, on 200 adult patients with psoriasis divided into two groups: (i) group 1, including 100 patients treated with narrow-band UVB phototherapy (nb-UVB), and (ii) group 2, including 100 patients treated with anti-TNFα. The patients included in group 2 had to be naive to cDMARDs and biologics and treated with anti-TNFα continuously for 5 years without loss of efficacy. All patients were observed for 5 years and underwent annual dermatologic examinations to assess for the occurrence of BCC or SCC. **Results:** A total of 34 out of 100 patients treated with phototherapy had one BCC or one SCC and 10 out of 34 developed two skin cancers. In particular, five had both types (one BCC and one SCC), and five had two BCCs. **Conclusions:** The results of our study highlight how the risk of developing NMSCs is greater in patients undergoing phototherapy compared to those treated with anti-TNFα. It also draws attention to the consideration that patients with scalp psoriasis might need closer follow-up as they could be more at risk of developing NMSCs.

## 1. Introduction

Psoriasis is a chronic, inflammatory skin condition that affects millions of people worldwide. An estimated two to three percent of the world’s population is affected by psoriasis, making it one of the most common skin diseases [1]. Epidemiologically, psoriasis makes no geographic distinction and can occur in individuals of any age, although onset is most common between the ages of 15 and 35 and between 50 and 65 [2,3]. While its exact cause remains unknown, a combination of genetic predisposition and environmental triggers is believed to play a crucial role in its pathogenesis [4]. In patients with psoriasis, the immune system mistakenly attacks healthy skin cells, accelerating the process of cell renewal. This leads to excessive production of keratinocytes, causing the formation of skin lesions characteristic of psoriasis, such as red, scaly plaques. Some studies indicate that the presence of certain genetic polymorphisms, such as HLA-Cw6, may increase the risk of developing the disease [4]. Close to this first mechanism, it is supposed that psoriasis could be related to an interaction between dendritic cells (DCs), hyperproliferative keratinocytes, mast cells, neutrophils, and T-cells [5,6]. The critical role of the immune system in psoriasis pathogenesis was corroborated when the administration of immune-suppressive agents, such as cyclosporine, proved to be successful in disease amelioration [7]. Initially, the pathogenesis was thought to be based on the overexpression of interferon (IFN)-γ and interleukin (IL)-12 signaling, with both CD4+ and CD8+ IFNγ-producing T-cells as key players [8]. Additionally, psoriatic skin was found to be infiltrated by TNFα- and inducible nitric oxide synthase (iNOS)-producing DCs that polarize T-cells to T helper-1 (Th1) and Th17 pathways [9]. More recently, the role of specific subsets of immune cells and their derived products has been established, such as the IL-23/Th17 axis [10]. Treatment options for psoriasis are various and depend on the severity of symptoms. Topical treatments, such as corticosteroid creams or vitamin D analogs, are often prescribed for mild cases. For moderate or severe forms, systemic treatments may be recommended and may include conventional disease modifying anti-rheumatic drugs (cDMARDs) or biologic agents that aim to modulate the immune response [11,12]. Psoriatic arthritis (PsA), depression, obesity, and cardiovascular diseases (CVDs) are considered common comorbidities. In addition, metabolic syndrome (MetS), hypertension, dyslipidemia, fibromyalgia, obesity, and atherosclerosis present a higher prevalence in recent years in psoriatic patients [13,14].

### 1.1. Psoriasis and Skin Cancers

An important aspect to consider in the management of psoriasis is the potential risk of developing skin cancers. This risk indeed represents a growing concern, as skin neoplasms are one of the most common forms of cancer globally [15]. The epidemiology of skin cancers is complex and varies depending on geographic, demographic, and environmental factors. According to World Health Organization (WHO) estimates, there are more than 2 million new cases of basal cell carcinoma (BCC) and squamous cell carcinoma (SCC), classified as non-melanoma skin cancers (NMSCs), each year [16]. These tumors, although rarely fatal, can result in significant impacts on patients’ quality of life and psychological health. The main cause of skin cancers is excessive exposure to ultraviolet (UV) radiation from the sun. The incidence of these cancers is significantly higher in regions with intense sun exposure, and people with fair skin or phototype I according to Fitzpatrick are particularly vulnerable. UV damages the DNA of skin cells, increasing the risk of genetic mutations that can lead to cancer formation [17]. However, not only sun exposure contributes to the risk. Other risk factors include tanning lamp use, the presence of many freckles or moles, immunosuppression, a family history of skin cancer, and age. Genetic predisposition plays a significant role in susceptibility to skin cancers. Indeed, individuals with a family history of malignant melanoma (MM) or other skin cancers may have an increased risk [18]. Some inherited conditions, such as xeroderma pigmentosum syndrome, impairing the ability of cells to repair DNA damage caused by UV exposure, greatly increase the risk of developing skin cancers.

### 1.2. Relation between Psoriasis, Skin Cancers, and Exposome

The exposome encompasses all environmental exposures, both infectious and noninfectious, that may contribute to disease onset. It posits that everyone’s disease, including psoriasis, is influenced by their unique history of exposures, in conjunction with their genetic susceptibilities [19]. Beyond traditional environmental factors such as air pollution and sunlight exposure, as well as lifestyle choices like diet and exercise, the exposome concept also includes psycho-social practices. Its outcomes, such as epigenomics, transcriptomics, proteomics, and metabolomics, are being spotlighted as potential disease mechanisms [20]. Oxidative stress conditions arise from an imbalance between the production and accumulation of oxygen reactive species (ROS) in cells and tissues, as well as the body’s enzymatic and non-enzymatic mechanisms to detoxify these reactive substances. Excessive oxidative stress results in the modification of various cellular components such as membranes, lipids, proteins, lipoproteins, and DNA, leading to the formation of toxic and mutagenic products [21]. UV radiation, air pollution, toxic substances, and their metabolites are responsible for generating reactive oxygen and nitrogen species (ROS/RNS). Skin and systemic oxidative stresses have been implicated in numerous skin diseases, including non-melanoma skin cancer [22].

### 1.3. Relation between Psoriasis, Skin Cancers, and Lifestyle

The use of tanning beds, for example, has been linked to an increased risk of melanoma, particularly in young people [23]. Awareness of the risks related to sun exposure and the promotion of sun protection practices are key to mitigating this growing problem. The aging population is another factor contributing to the risk of skin cancer. As we age, the skin becomes less able to repair the damage caused by exposure to the sun over the years, increasing the likelihood of developing skin cancer. In detail, in patients over than 65 years, the inadequate response to UV-induced damage appears to be mainly due to an increased rate of senescent fibroblasts in the papillary dermis with overexpression of the onco-suppressor p53 inactivating protein (BP53) [24]. This would lead to a failure to block the cell proliferation of mutated clones and, thus, tumor progression. This underscores the importance of constant surveillance, especially in older people, for early detection and timely treatment. Epidemiological studies have highlighted a link between psoriasis and an increased risk of NMSCs and MM. Indeed, persistent skin inflammation associated with psoriasis may contribute to carcinogenesis by increasing cell proliferation and impairing the normal DNA repair process [25]. It is well established that some cancers are influenced by chemical mediators that increase the production of reactive oxygen species (ROS) and alter the timing of the proliferation, differentiation, and maturation of keratinocytes, as also evidenced in psoriasis [26,27]. Therefore, patients with psoriasis should be aware of this risk and take preventive measures, such as regular skin examination and sunscreen, to reduce the likelihood of developing skin cancers.

### 1.4. Aim of This Study

Considering phototherapy on one hand, which exerts a direct damaging effect on keratinocytes [28], and immunosuppression induced by anti-TNFα agents on the other [29], patients with psoriasis may be exposed to two of the best-known risk factors for developing NMSCs during their lifetime.

In previous studies, patients treated with biologic therapies did not show a direct correlation with higher skin cancer risk compared to those treated with non-biologic systemic therapy [30]. However, it was not possible to exclude bias related to previous treatments undertaken, including phototherapy.

Indeed, the extent to which two distinct risk factors, immunosuppression induced by anti-TNFα agents and phototherapy, may individually affect the risk of developing NMSCs in psoriatic patients has not been entirely assessed.

The aim of our study was to evaluate the risk of developing NMSCs in psoriatic patients with a disease duration of less than 6 years, observed for at least 5 years from January 2018 to January 2023, by directly comparing patients only treated with phototherapy and patients treated with anti-TNFα agents, naive to other systemic treatments or phototherapy.

## 2. Materials and Methods

This study was conducted under the 1964 Declaration of Helsinki and all subsequent amendments. We conducted a single-center retrospective study at Siena University Hospital, Italy, on 200 adult patients with psoriasis divided into two groups: (i) group 1, including 100 patients treated with narrow-band UVB phototherapy (nb-UVB, wavelength 311–313 nm), and (ii) group 2, including 100 patients treated with anti-TNFα. Eligible patients were aged ≥ 18 years and had been diagnosed with psoriasis with a disease duration of at least 6 years. Patients included in the first group underwent phototherapy for 5 years, without application of topical steroids or vitamin D analogs, on a schedule of 2 sessions per week for 8 weeks with a 3-month break between the two treatment cycles (16 sessions per cycle, 32 sessions annually, 160 sessions total). The cumulative dose was 8750 mJ/cm^2^ per session (global cumulative dose for patient: 87,500 mJ/cm^2^). An assessment of minimal erythemal dose (MED) for individual patients was used to guide the nb-UVB phototherapy. To minimize possible bias, selected patients should not have concomitant diseases or be on systemic drugs that could potentiate the UV kinetics and thus facilitate the development of NMSCs. The patients included in group 2 had to be naive to cDMARDs and biologics and treated with the anti-TNFα agent adalimumab as a subcutaneous injection, administered at the starting dose of 80 mg, followed by 40 mg after a week and then 40 mg taken every 2 weeks continuously for 5 years without loss of efficacy. To be included in this study, the patients in group 2 had to have never undergone nb-UVB phototherapy. Patients who reported a loss of efficacy or in whom the use of topical therapies or cDMARDs was necessary were excluded from this study. We selected patients treated with anti-TNFα and not with other biologic agents (anti-IL-17 or anti-IL-23), since anti-TNFα agents are indicated in the regional guidelines as first-line drugs in patients with moderate-to-severe psoriasis. According to our outpatient practice, we selected patients receiving biosimilar adalimumab who constitute the largest proportion of patients currently managed. All patients were observed for 5 years and underwent annual dermatologic examinations to assess for the occurrence of BCC or SCC. Patients who developed MM were excluded from this study and observation. Patients with a previous history of use of tanning beds were excluded from this study. Patients were defined as obese if their BMI was >30.

### Statistical Analyses

Descriptive statistics were acquired. The mean and standard deviation were estimated for quantitative variables, while absolute frequencies and percentages were calculated for the qualitative ones. A chi-squared test was performed to compare qualitative variables with phototherapy and anti-TNF groups. Student’s *t* test was performed to compare ages between the two groups. Kaplan–Meier curves were estimated, and a log-rank test was performed to compare the onset of BCC, SCC, or combined events (BCC-SCC), in patients treated with phototherapy or anti-TNFα. Univariate and multivariate Cox regression were carried out to estimate Hazard Ratios (HRs) and their 95% confidence intervals (95% CIs). A *p*-value < 0.05 was considered statistically significant. All the analyses were carried out using R software, version 4.3.1.

## 3. Results

### 3.1. Phototherapy and Anti-TNFα

A total of 200 patients were included in this study. The two groups, phototherapy and anti-TNFα, were comparable in terms of age, sex, and involvement of difficult-to-treat areas. Obesity was the most frequent comorbidity in our patient cohort; however, no statistically significant difference was found in terms of obese patients between the phototherapy and anti-TNFα groups. The two groups were compared directly, without a control group, and included patients who had not previously been exposed to other medications to eliminate possible confounding factors to directly correlate the risk of the therapy given. Clinical and demographic characteristics of the patients are shown in Table 1. In total, 34 out of 100 patients treated with phototherapy had one BCC or one SCC, and 10 out of 34 had two tumors. In detail, five developed both skin cancers (one BCC and one SCC), and five had two BCCs.

### 3.2. Survival Analysis

**Figure 1:**
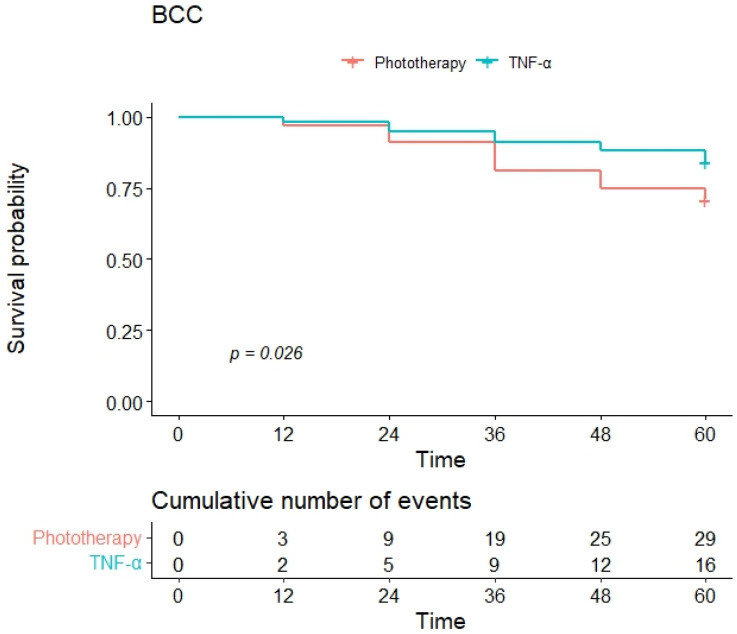
Kaplan–Meier curves of event-free survival for BCC as the intercurrent event.

**Figure 2:**
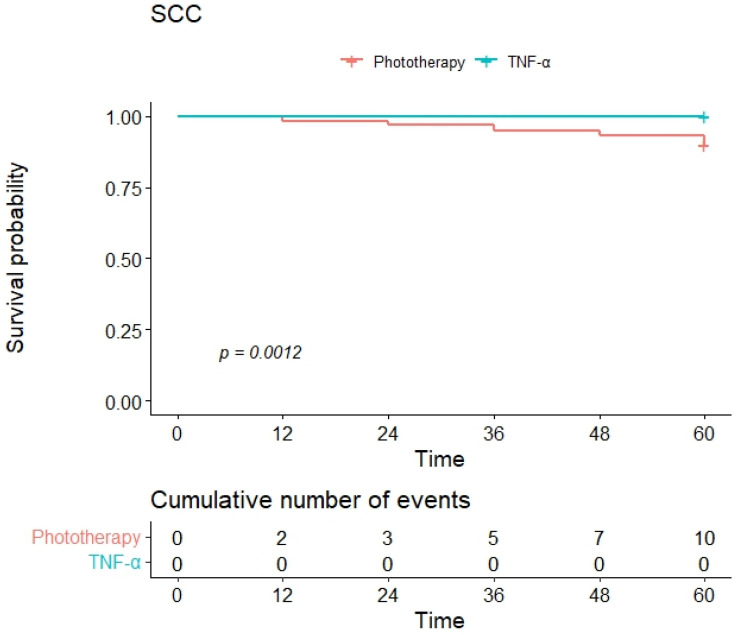
Kaplan–Meier curves of event-free survival for SCC as the intercurrent event.

**Figure 3:**
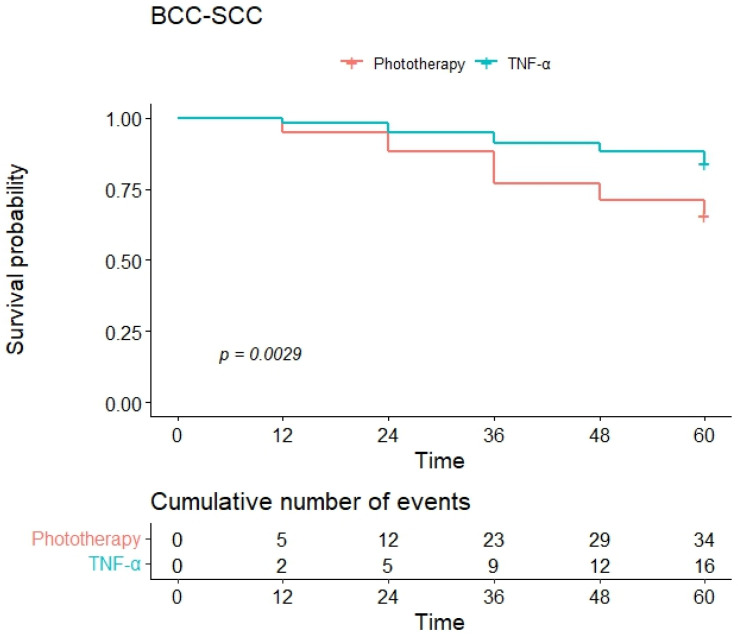
Kaplan–Meier curves of event-free survival for either BCC or SCC as intercurrent events.

Event-free survival in both groups was examined using Kaplan–Meier survival analysis for the following intercurrent events: occurrence of a BCC, of an SCC, and of at least one of the two (BCC-SCC). When considering the development of BCC as the intercurrent event, the Kaplan–Meier curves showed a one-year event-free survival of 97% and a four-year survival of 75% in the phototherapy group and 98% and 88% in the anti-TNF group (Figure 1). Furthermore, when considering the development of SCC as the intercurrent event, the group of patients treated with anti-TNFα had better event-free survival, and there were no cases of SCC in this group. In detail, one-year and four-year event-free survivals were 100% in the anti-TNF group and 98% and 93% in the phototherapy group (Figure 2). When considering the occurrence of either a BCC or an SCC (BCC-SCC) as the intercurrent event, the one-year event-free survival was 95% in the phototherapy and 98% in the anti-TNF group (Figure 3). At four years, event-free survival was 71% and 88% in the phototherapy and anti-TNFα groups, respectively. The HR of the univariate Cox regression for the occurrence of either a BCC or an SCC (BCC-SCC) were analyzed and are reported in Table 2. From the analysis, therapy with anti-TNFα agents, age, and scalp psoriasis were the only significant factors correlated with event-free survival. In detail, the use of anti-TNFα was predictive of a significantly better event-free survival versus phototherapy [HR of 0.42 (95% CI: 0.23–0.75)]. Age was significantly correlated with a worse one-year event-free survival [HR of 1.12 (95% CI: 1.08–1.15)]. Similarly, scalp psoriasis was significantly correlated with a worse event-free survival [HR of 1.97 (95% CI: 1.10–3.53)] (Table 2). These three variables were further included in a multivariate Cox regression analysis, showing that only age (HR: 1.12, 95% CI: 1.09–1.16, *p* < 0.001) and anti-TNFα treatment (vs. phototherapy) (HR: 0.28, 95% CI: 0.15–0.51, *p* < 0.001) significantly correlated with worse and better event-free survivals, respectively.

## 4. Discussion

In the vast and intricate landscape of dermatological research, our investigation delved into the factors that may determine the risk of developing NMSCs in patients with psoriasis, as well as the intricate interplay between putative risk factors and therapeutic interventions. Our study centers its attention on a comparative analysis, drawing a dichotomy between cohorts of patients undergoing either phototherapy or therapy with anti-TNFα agents. In the literature, there are conflicting opinions about the risk of developing NMSCs in patients with psoriasis, especially regarding the difficulties of completely detailing a patient’s daily habits at the time of observation and prior to this. In one study conducted by Paradisi et al., psoriasis patients had a 16% decreased risk of developing NMSC when compared to those without dermatological disorders [31]. According to recent meta-analyses, individuals affected by psoriasis are more susceptible than the general population to developing NMSCs. In detail, one meta-analysis demonstrated that patients with psoriasis had a 1.72-fold higher risk of developing NMSC in comparison to non-psoriatic patients (RR, 1.72, 95% CI: 1.46 to 2.02). Furthermore, patients with moderate to severe psoriasis had a higher risk of NMSC (RR, 1.82, 95% CI: 1.38 to 2.41) than those with mild psoriasis (RR, 1.61, 95% CI: 1.25 to 2.09) (*p* < 0.001) [32]. Frequently, people treated with biologic treatments for psoriasis had previously used phototherapy and/or cDMARDs, both of which may enhance the risk of skin tumors, highlighting how difficult it may be to discriminate the real risk of developing NMSCs in these patients [33]. The results have typically been interpreted in the context of exposure to various systemic treatments, with a focus on 8-methoxypsoralen plus ultraviolet A (PUVA) therapy, which can cause p53 mutations and promote the emergence of NMSC in psoriasis patients [34].

In our study, the results prompted an increased risk of developing SCC and/or BCC in patients receiving phototherapy vs. anti-TNFα agents, along with the absence of SCC in patients treated with anti-TNFα. In addition, our study selectively focused on patients who had not undergone previous therapies with MTX or other cDMARDs, further corroborating these results.

The risk of developing NMSCs under anti-TNFα therapy is indeed still incompletely clarified. In the BADBIR (Biologics and Immunomodulators Register), no associations are reported between the occurrence of BCC or SCC and biologic agents, in comparison to non-biologic systemic therapies [35]. In total, 267 (2%) patients with a history of BCC or SCC (190 with previous BCC only; 62 with previous SCC only; 15 with previous BCC and SCC) were identified, and 14,800 (98%) patients were included in the BADBIR cohort study for first incidence of BCC or SCC. Patients with a history of BCC or SCC were more likely to have Fitzpatrick skin type 1 (20% vs. 12%) and were significantly older with a longer duration of psoriasis, and a higher proportion was previously treated with acitretin (46% vs. 37%) or PUVA (36% vs. 24%). In total, 32 patients (12%; 21 biologic cohort; 11 non-biologic systemic cohort) with a history of BCC or SCC developed an incident BCC, and 28 patients (11%; 19 biologic cohort; nine non-biologic systemic cohort) developed an incident SCC [35]. An observational study (Psoriasis Longitudinal Assessment and Registry (PSOLAR)) was conducted on 12,000 psoriasis patients who underwent continuing phototherapy or systemic therapy [36]. All patients had an 8-year follow-up, and no one had a history of BCC or SCC. The results demonstrated a markedly elevated risk of developing BCC, but not SCC, in the TNFα-inhibitor and methotrexate (MTX) groups. Notably, the elevated risk of NMSC in psoriasis appeared to be unrelated to the severity of the condition or the presence or absence of arthritis [36].

Whether it is well established that chronic exposure to UV radiation can directly damage keratinocytes, the biological events behind a supposed NMSC development following TNFα antagonists are not settled. It is known that TNFα is involved in apoptotic events and in the regression of neoplasms. However, the molecular events are notoriously diverse and complex, probably involving the NF-kB pathways and the kinetics of cancer progression [37]. Furthermore, TNFα does play important functions in immunosurveillance for cancer cells. Some studies indeed suggest that TNFα can counteract carcinogenesis, as well as that exposure to TNFα inhibitors might be associated with an elevated risk of developing an NMSC [37,38].

Interestingly, a study compared the risk of developing NMSCs under anti-TNF-α agents in patients with psoriasis and rheumatoid arthritis (RA). In this study, the time to occurrence of the first NMSC after the initiation of TNFα inhibitors was shorter and the rate of NMSCs was higher in psoriasis patients compared to RA patients. The total event rate was 5.5 (2.2–13.4 95% CI) times higher in the psoriasis group [39]. It is therefore possible that disease-related factors like previous phototherapy could have influenced the results. This was supported by the finding that the proportion of patients diagnosed with NMSCs within the first year after the start of anti-TNFα agents was found to be higher in psoriasis (36%) compared with RA (17%) [39]. Our data underscore a higher risk of developing both BCC and SCC among individuals undergoing phototherapy versus patients treated with anti-TNFα agents. Particular attention should be paid to the possibility of the double incident event (BCC-BCC or BCC-SCC or SCC-SCC). In our study, only ten patients developed a composite event, all in the phototherapy groups: in detail, five patients had a double BCC and five patients had both. No patients treated with anti-TNFα agents reported any composite event. Thus, it appears from this study that although the mechanism correlating the occurrence of NMSCs in anti-TNFα-treated patients is not completely clear, it is likely related to immunosuppression secondary to previous treatments and the combined action with phototherapy.

Pulling together the variables that may influence NMSC risk, age emerges as a risk factor, with individuals aged more than 65 exhibiting a conspicuous vulnerability to NMSCs. The recognition of age-related susceptibility emphasizes the need for nuanced, age-tailored treatment plans and vigilant monitoring of psoriasis patients, including, for example, the avoidance of phototherapy in elderly patients. Furthermore, our analysis suggests that one of the well-known difficult-to-treat sites of psoriasis, the scalp, may somehow be correlated with the occurrence of an NMSC. Indeed, our investigation highlights a higher frequency of NMSCs in patients affected by scalp psoriasis in both the phototherapy and anti-TNFα groups (HR: 0.51, *p* = 0.023). This unexpected association with scalp psoriasis prompts reflections on the effects of treatment on specific anatomical regions. This finding should encourage further investigation into the possible factors that might make this region a predictor for the development of NMSCs. Furthermore, the results of our study show the importance of carefully considering each individual psoriasis patient and possibly weighing up the choice of treatment in patients with scalp psoriasis, given the increased risk of developing NMSCs. Regarding other possible comorbidities or disease localization, our study did not report any correlation with the occurrence of NMSCs that seems to be independent from disease severity or the presence or absence of psoriatic arthritis.

Limitations of this study might be related to the observation time, which should be longer, especially in patients treated only with phototherapy. In addition, although subjected to continuous monitoring, it is not possible to say with certainty that patients did not independently apply local steroids without notifying the investigator or even controlling photoexposure during the summer period.

Future research should be encouraged to better understand the molecular, genetic, and immunological factors underpinning the observed patterns.

## 5. Conclusions

The relationship between inflammation and the development of malignancies in psoriasis patients has not been fully explored. The incidence of skin cancer in people with psoriasis, particularly NMSCs, has been the subject of numerous publications in recent decades, including studies of the literature, peer-reviewed studies, and meta-analyses, with a range of findings and recommendations [40]. Systemic UV therapies that can be used to treat psoriasis may enhance the risk of malignancy in psoriasis-affected individuals. The comorbidities of psoriasis, such as alcohol consumption, smoking, and overexposure to the sun, can also worsen the chronic inflammatory state and make it more difficult to respond to immunosuppressive drugs. As per the reports, these factors are thought to be the main reasons for the greater prevalence of skin tumors. Despite efforts in psoriasis management, a multidisciplinary approach involving dermatologists and other health professionals is essential to ensure comprehensive and individualized care. Patient education is equally critical, as a thorough understanding of the disease can improve treatment compliance and overall quality of life. As it emerges from our study, it is important to perform skin checks on patients prior to starting biologics and to have increased vigilance in skin cancer surveillance during treatment. This is especially important in patients with known risk factors such as older age, a history of previous skin cancers or actinic damage, a family history of skin cancers, and previous immunosuppressive therapy or phototherapy.

As we navigate the complex terrain of dermatological interventions, the results of our study can help clinicians navigate the maze of risks and benefits as well as the need for individualized patient care in psoriasis.

In conclusion, our study represents an important contribution to the evolving narrative on NMSC risk associated with dermatological interventions. The intersection of treatment modalities, age-related vulnerabilities, and anatomical location forms a picture of complexity that encourages a more complete and precise understanding of this delicate subject.

## Figures and Tables

**Table 1:** Clinical and demographic characteristics of the patients.

Clinical and Demographic Characteristics	Phototherapy	Anti-TNF	*p*
N	100	100	
Gender = M (%)	63 (63.0)	61 (61.0)	0.884
Age (mean (SD))	55.06 (14.98)	58.47 (12.27)	0.080
Positive family history_BCC_SCC = 1 (%)	16 (16)	11 (11)	0.303
BMI = obese (%)	13 (13.0)	8 (8.0)	0.356
Psoriatic arthritis = YES (%)	12 (12.0)	20 (20.0)	0.177
Nail psoriasis = YES (%)	26 (26.0)	36 (36.0)	0.169
Palms/soles = YES (%)	34 (34.0)	38 (38.0)	0.659
Scalp = YES (%)	43 (43.0)	53 (53.0)	0.203
Genitals = YES (%)	19 (19.0)	23 (23.0)	0.602

**Table 2:** Univariate Cox regression analysis.

Variables	HR	95% CI	*p*-Value
Male	0.85	0.49, 1.50	0.58
Age	1.12	1.08, 1.15	<0.001
Familiarity per NMSC	0.50	0.18, 1.40	0.19
Obesity	0.97	0.38, 2.43	0.94
Arthritis	1.47	0.75, 2.88	0.26
Nail psoriasis	1.06	0.58, 1.92	0.85
Palms/soles	1.20	0.68, 2.11	0.53
Scalp	0.51	0.28, 0.91	0.023
Genitals	0.58	0.26, 1.29	0.18
Anti-TNF therapy	0.42	0.23, 0.75	0.004

## Data Availability

The data that support the findings of this study are available from the corresponding author upon reasonable request.
